# A two-pronged approach to understanding reciprocity and mental health relationship in developing countries: evidence from young informal construction workers in Nigeria

**DOI:** 10.1186/s12889-024-19315-x

**Published:** 2024-07-11

**Authors:** Ayomide Oluwaseyi Oladosu, Timothy Chanimbe

**Affiliations:** 1 School of Graduate Studies, Lingnan University, 8 Castle Peak Road, Tuen Mun, Hong Kong; 2 Government and International Studies, Hong Kong Baptist University, Baptist University Road, AAB 11/F, Kowloon Tong, Hong Kong

**Keywords:** Mental health, Young people, Reciprocity, Social support, Informal sector, Developing countries, Nigeria

## Abstract

**Background:**

Mental health problems disproportionately affect young people in developing countries. However, there is limited research on help-seeking behaviours and the social support systems that improve mental wellbeing among vulnerable youth populations.

**Objective:**

This mixed-methods study aimed to examine the relationship between social support reciprocity and mental health among young informal construction workers in Nigeria, a population at high-risk for occupational and socioeconomic stressors.

**Methods:**

A cross-sectional survey was administered to 686 informal workers to measure reciprocity, mental health-related quality of life, and covariates. In-depth interviews with 32 participants provided qualitative context.

**Results:**

Quantitative analyses showed 25% of participants reported poor mental health. Reciprocity positively predicted mental health after controlling for covariates. Qualitative findings revealed reciprocity occurs directly between individuals as well as indirectly through trade unions and religious groups. Indirect exchanges through groups helped address limitations of direct support due to limited resources.

**Conclusions:**

This study fills important gaps in understanding how social relationships impact mental health in developing country contexts. Findings emphasize the role of collective action and community-based support systems in promoting mental wellbeing among vulnerable populations. Insights can inform culturally relevant, systems-level mental health interventions.

**Supplementary Information:**

The online version contains supplementary material available at 10.1186/s12889-024-19315-x.

## Introduction

The portrayal of health as the greatest wealth of humans has unsurprisingly proven its worth having featured prominently as the third spine of the Sustainable Development Goals (SDGs 3), emphasizing healthy lives and the promotion of well-being for all at all ages [57]. The SDG 3.4’s accentuation and underscoring of mental health is a warning sign for global alertness [52]. Apparently, there has been an emerging interest in the mental health of young people among scholars and practitioners alike. Despite the high rate of mental health problems among the youth, discourses regarding the modus of help-seeking vis-à-vis the use of support resources to improve mental health have been relatively sparse [46, 65]. The United Nations and its subsidiaries which categorized people between the ages of 15 to 24 as youth [56], also disclose how mental health challenges are rife within this age group [58]. On a global scale, one in seven adolescents of 10–19 years battle some form of mental disorder, which accounts for 13% of the global burden of disease in this age category. Suicide has therefore been ranked as the fourth cause of death among young people of 15–29 years [68].

As UNICEF [55] unveils, there is a wide gap between mental health funding and the mental health needs of young people. It emerged that governments allocate only 2.1% of their health expenditure to mental health and the economic price the global economy pays for this neglect is losing approximately USD387.2 billion of human potential that could have been invested in national economies annually. Gauging from the rapid proliferation of mental health problems within a teeming population of approximately 1.2 billion who identify as youth and the uncaptured ages of 25 to 29 [54], a sizeable fraction of the global population and a valuable asset to the global economy could be lost through this medical condition. Particularly in the context of COVID-19, there was also a massive global decline in youth mental health. The findings of Simon-kumar & Gluckman [46] reveal that prior to the onset of the COVID-19 pandemic, there were rising rates of suicidal tendencies and behaviour as well as increasing rates of depression, and a general decline in emotional wellbeing among the youth. More acutely, the COVID-19 pandemic led to even higher levels of social isolation, anxiety and stress. The pandemic exacerbated youth mental health problems after having penetrated the constantly changing labour market, increasing unemployment and underemployment along with an upsurge in obscene occupations [23]. The pandemic aggravated the already numerous labour market challenges faced by young people. Over the period of 2019–2020, ILO reports a higher rate of job losses among the youth than adults [23].

For employed youths, the considerable amount of manual dexterity and poor working environment are significant factors influencing the mental health of employed youths [45, 67]. In 2019, 15% of workers worldwide experienced mental health issues, resulting in 12 billion lost working days and a cost of USD 1 trillion annually in lost productivity [67]. Distinctively, the environment and accompanying tasks for informal jobs are usually hazardous, and precarious along with meagre and unstable wages [19, 22]. Additionally, these jobs typify the absence of social protection and compensatory benefits, such as pensions, health insurance, or leave [19, 22]. Due to the porousness of informal jobs in the developing world, Silva-Peñaherrera et al. [45] find employees in informal jobs with a higher prevalence of poor mental health than their counterparts in formal jobs. Studies have shown a higher prevalence of poor mental health among informal job workers compared to those in formal employment [18, 45]. In Africa, misconceptions about mental health and neglect of young people's emotional and psychological well-being contribute to a false perception of their overall health [5].

Perming the above with estimations that 77% of young people work in the informal sector [36, 47], it is not out of place to draw conclusions that young informal workers are the most preyed by mental health problems [4]. This then begs the question, how do young informal workers improve their mental health? Scholars have demonstrated how young informal workers rely on the support of their social networks to meet their mental health needs [39, 46, 65]. The extant studies theorize how young people rely on reciprocity in social networks to assist one another in improving their mental health (*see for example*, [24, 25, 49, 50]). These studies hypothesized young people as agents building intimacy and distanciation in which they are developing their own identity as well as reciprocal relationships with key people in their social milieu. Hence, young people with established relationships where social support is reciprocal are likely to report better mental health outcomes than their counterparts in less established reciprocal relationships (*see for instance* [24, 49])). However, the prevailing literature that delves into how reciprocity in social networks improves the mental health of young informal workers has been inadequately discerned across the globe.

The existing literature on reciprocity within social networks has predominantly focused on developed countries, overlooking the intricate dynamics prevalent in the developing world, where labour informality is widespread. This study aims to bridge this gap by investigating the relationship between reciprocity and the mental well-being of young workers in Africa. It delves further into the exploration of diverse forms of reciprocity within social networks, which play a significant role in shaping the mental health outcomes of young informal workers in Africa. While prior research has characterized reciprocity as a direct exchange of social support between individuals, we argue that this direct person-to-person exchange may be imbalanced in the developing world context due to the substantial economic constraints faced by many young informal workers. Consequently, this imbalance can have adverse effects on their mental health. Thus, we propose that cultivating balanced reciprocal relationships among young informal workers would necessitate indirect channels such as active participation in groups with shared interests (such as, religious associations or trade unions), where resources are pooled together to support fellow members. By embracing this indirect reciprocal approach, rather than relying solely on direct person-to-person exchanges, we anticipate a positive impact on the mental health of young informal workers.

The significance of these geographical and theoretical questions demand that we draw data from Nigeria to provide empirical evidence from Africa to first test claims of a relationship between reciprocity in social networks and young informal workers’ mental health. Secondly and more importantly, we delve deeper to provide thick description and evidence of the kinds of reciprocity in social networks and their impact on the mental health of young informal workers. Nigeria, with a population of about 224 million [69], faces high youth unemployment rates ranging from 42.5% to 52.5% [30, 69]. This has led to a significant rise in informal employment, which accounts for 92.9% of the country's workforce [21]. Construction workers, in particular, face significant mental health challenges due to the hazardous and stressful nature of their jobs [34]. Therefore, it is essential to examine how reciprocity within social networks supports the mental health of young informal workers in Nigeria.

Policy wise we expect the results of this study to be significant for practitioners, particularly from the less developed countries interested in improving the mental health of young people. The rest of this paper will present the following sections/sub-sections: the conceptual relationship between reciprocity and health, the research method employed in this study, the data analysis, the results, the discussion of the findings, and the conclusion.

### Silent struggles: unveiling the mental health burden of young informal construction workers in Nigeria

The construction industry in Nigeria is a significant employer of labour, engaging approximately 20% of the working population, with a majority of workers employed informally [31, 35, 38]. Within this industry, formal workers, such as Engineers and Architects, primarily assume supervisory roles, while informal workers, commonly referred to as artisans, tradesmen, or labourers, carry out the labour-intensive tasks on construction sites [37]. Given the physical demands of construction work and the escalating youth unemployment rate in Nigeria, young people constitute a substantial portion of the workforce in this industry. Informal workers, in particular, make up the largest working population, accounting for approximately 55% to 89% of the industry’s workforce [8, 21].

In the bustling construction sites of Nigeria, where dreams of towering structures take shape, a silent battle rages within the hearts and minds of young informal construction workers. Despite the construction industry being the most hazardous among all sectors in Nigeria, responsible for approximately 40% of work-related accidents [11, 33], the mental health challenges faced by these workers have largely remained overlooked. This section delves into the untold struggles of young informal construction workers, shedding light on the profound mental health challenges they endure in the demanding world of informal construction. From the lack of job security and economic hardships to unsafe working conditions and social marginalization, their struggles encompass a myriad of complex issues that deeply impact their well-being.

Many young construction workers in Nigeria find themselves trapped in the informal sector, devoid of the benefits and protections (like health insurance, pension, leave, formal contracts, and stable income) associated with formal employment. Their livelihoods hinge on day-to-day arrangements and short-term contracts, leaving them vulnerable to constant job insecurity and a perpetual cloud of uncertainty regarding their future income. This weight of this instability possibly seeps into their psyche, potentially amplifying issues of anxiety, stress, depression, and other mental health concerns. Additionally, within the realm of informal construction, meagre wages perpetuate a cycle of economic hardship for young workers. Their earnings often fall far below what is necessary to meet their basic needs, let alone access proper housing, healthcare, education, and other essential services. This financial strain becomes a constant companion, casting a shadow over their mental well-being and limiting their capacity to envision a brighter future.

Furthermore, the construction sites that serve as the backdrop for their toil are riddled with hazards and devoid of adequate safety measures [16, 20, 26, 34]. Young construction workers brave these perilous environments, lacking proper protective equipment, comprehensive training, and sufficient supervision. The constant exposure to accidents, injuries, and long-term health issues breeds a sense of fear and vulnerability, which may further exacerbate their mental health challenges [12, 34].

Again, construction work is an unyielding test of physical endurance, demanding long hours of labour-intensive tasks. Young workers, often sacrificing their well-being for survival, endure backbreaking labour, heavy lifting, repetitive motions, and exposure to extreme weather conditions. The toll on their bodies leads to fatigue, physical exhaustion, and heightened susceptibility to injuries, which, in turn, can burden their mental resilience. Despite all these, informal construction workers in Nigeria are deprived of critical social protection measures such as healthcare, retirement benefits, and unemployment benefits [36]. This lack of support exacerbates their financial insecurity, making it even more challenging to cope with emergencies or unexpected events. The absence of a safety net intensifies their mental strain, which potentially leaves them disheartened and devoid of hope.

For many young informal construction workers, educational opportunities and skills training are elusive dreams. The absence of quality education and skill development programs hampers their ability to secure higher-paying and more stable employment in the future. This limited access stifles their potential, perpetuating a cycle of low wages and further curtailing their mental well-being.

In the light of all these, mental healthcare is still not a common phenomenon in Nigeria and is often associated with insanity [2]. For instance, it was only in January 2023 did a new mental health law in Nigeria change from the ‘lunacy ordinance’ to mental health act. This situation has led to increased stigma and isolation to the victims of mental problems in the country. Such stigmatization, perceptions and belief of mental health conditions make it difficult for young informal construction workers to report mental health cases or seek mental healthcare. It is only recently, particularly after the emergence of COVID-19 did mental health awareness increasingly gain popularity in the Nigerian context, particularly caused by the limitations on social interactions from social policies enacted by the government to reduce the spread of COVID19 [3].

Furthermore, mental health services in Nigeria are limited in supply and often come at a high cost, rendering them accessible primarily to individuals of certain socioeconomic class who can afford it. Consequently, underprivileged groups such as young informal construction workers are effectively excluded from availing themselves of biomedical interventions for addressing mental health problems. This limitation underscores the need for a comprehensive approach to mental healthcare, as advocated by the World Health Organization (WHO) through its social determinants of health framework [10, 43, 66]. Recognizing the challenges posed by limited access to biomedical measures, the WHO has embraced a holistic approach to mental health, encompassing non-biomedical strategies that leverage social networks. In line with this perspective, the WHO has adopted the Comprehensive Mental Health Action Plan 2013–2020 [42]. This plan recognizes the importance of integrating both biomedical and non-biomedical approaches in addressing mental health problems, particularly in low and middle-income countries (LMICs) like Nigeria. By embracing a diverse range of interventions, this approach aims to bridge the gaps in mental healthcare and promote equitable access to support for individuals across different socioeconomic backgrounds. Hence, the aim of this study is to examine the impact of social support reciprocity on the mental health of young informal construction workers in Nigeria.

### Reciprocity and mental health

Reciprocity refers to a “give-and-take” process that establishes stable social relationships in an individual’s life [14]. It is considered the fundamental unit of social relationships, which involves responding to positive actions with positive actions, such as rewarding kind behaviour [29]. In professional helping relationships, reciprocity is seen as a process of exchanging emotions or services and is widely recognized as a central aspect of human life [29, 60]. Reciprocity becomes balanced when individuals are satisfied with their contribution to the group, acknowledgment from other members, and benefits received from the group.

Researchers have linked reciprocity to mental health, suggesting that reciprocity in social relationships is generally associated with positive mental health. Sandhu et al. [41] in a systematic review found that incorporating reciprocity as a component of mental healthcare, with recurrent and observable processes may be harnessed to promote positive outcomes. Lack of reciprocity in social relationships was generally associated with negative effects and poor mental health. In a cross-sectional study, using the effort-reward balance model, von dem Knesebeck & Siegrist [60] investigated the potential imbalance between effort spent (investment in relationships) and rewards received (benefits from the relationship) among marital, parental, and unspecified relationships. The study comprised 1290 noninstitutionalized elderly men and women ≥ 60 years of age: 682 in Germany (mean age, 70.8) and 608 in the United States (mean age, 72.3). The study’s results revealed consistent associations of nonreciprocal social support with depressive symptoms for both genders (male and female) in both samples. The risk of depressive symptoms was about twice as high among elderly men and women who reported nonreciprocity in their social exchanges compared to subjects reporting reciprocal social exchange [60].

Furthermore, studies on the relationship between reciprocal social exchange for health and well-being tested the associations of different types of social activities (paid work, caring, and volunteering) and well-being [28], and between social productivity (voluntary or charity work, caring for a sick or disabled adult, and provision of help to family, friends, or neighbours) and well-being [62]. According to Siegrist et al. [44], socially-productive activities are based on the social norm of reciprocity, “…*in which the effort of doing the activity is made in anticipation of an equivalent reward that reflects the value of the effort involved.*” Data from a cross-sectional wave in 2004 of the English Longitudinal Study of Ageing (ELSA), of 5384 participants at post-state pension age (≥ 60 years for women and ≥ 65 years for men), were analysed to examine whether participation in social activities (i.e., caring for another person) was associated with higher levels of well-being (i.e., depression), and explained by “the reciprocal nature of these activities” [28]. The study showed that reciprocal exchange had a negative association with the degree of depression regarding the activity of caring [28].

Similarly, the cross-sectional study of Wahrendorf et al. [62] also investigated the relationship between social activity (defined as social productivity, i.e., caring for a sick or disabled person and provision of help to family) and well-being (i.e., depression), focusing on the quality of the activity based on the notion of exchange reciprocity. The study was accomplished on 22 000 participants, ≥ 50 and from ten European countries—using data from the SHARE study (“Survey of Health Aging and Retirement in Europe”). The study uncovered that reciprocal activity was associated with lower scores on depression both for caring and informal help. Thus, all three studies [28, 44, 62] are lending “…*support to Siegrist's observations on the importance of reciprocal exchange in social relations for health and wellbeing*” [28].

However, most of the existing research have focused on middle-aged and older individuals, leaving a gap in understanding the association between reciprocity and mental health among young people. When it comes to young people, there are few studies that have examined the relationship between reciprocity and mental health, and all of them are in developed countries. For instance, in Finland, Tuominen & Haanpaa (2022) used a cross-sectional design to explore the association between social capital (measured by reciprocity) of young people at 12–13 years and their subjective well-being using Finland’s sub-sample of the third wave of the International Survey of Children’s Well-Being. The study measured well-being with two context-free scales: a one-dimensional overall life satisfaction scale and a five-dimensional Student’s life satisfaction scale. The study found that norms of reciprocity was significantly associated with positive wellbeing among young people in Finland. Among the different social groups evaluated, reciprocity between family-related social networks had the greatest significance on young people’s wellbeing [53].

Another study conducted in England and Finland by Törrönen [49] used participatory action research method to examine how young adults in England and Finland who have been in the care system evaluate their social relationships and mental health and how these are interconnected. The interview data explored young adults’ well-being during the transition period from care to independent living. The study found that young adults who have supportive social networks report better mental well-being and security than those who do not have such networks. In general, the study found that reciprocal social relationships are important for developing social skills, keeping physically and mentally well, and feeling a sense of security [49].

In Japan, Jou & Fukada [25], examined the effects of reciprocity and sufficiency of social support on the mental and physical health of 488 Japanese university students with different levels of stressors. The authors examined the participants’ support relationships with others and found that reciprocity of support appeared to have both direct and buffering effects on health. The study found that lack of reciprocity of social support is associated with negative affect and poor health among Japanese university students. Reciprocity of support with family showed a direct effect on stress-related symptoms. Individuals in reciprocal relationships show better health than those in nonreciprocal relationships, whether stress is present or not. Overall, the article suggests that reciprocity and sufficiency of social support are important factors in maintaining good mental health among university students in Japan.

While the above studies highlight the importance of reciprocity on the mental health of young people, there remains significant lacunas in the reciprocity and mental health literature that requires immediate attention. First, all the studies cited above focused on young people in developed countries in Europe and Asia. This indicates a geographical/contextual gap in the understanding of the association between reciprocity and mental health among young people from developing countries, especially Sub-Saharan Africa (SSA), which is largely a youthful population. This is important because cultural and social norms of reciprocity, which may play a role in the mental health of young people from SSA, are very specific to the region and have not been studied in detail. Therefore, it is imperative to conduct further research in this area to understand how reciprocity and mental health are associated in this region. In light of this gap, this study aims to examine the relationship between social support reciprocity and the mental health of young informal construction workers in Nigeria. By focusing on this specific population, the study intends to contribute to the understanding of reciprocity and mental health in a developing country context and among underprivileged young people.

### Study aims and hypotheses

The following aims and hypotheses were framed for our study.

#### Aim 1

 To evaluate the mental health-related quality of life of young informal construction workers in Nigeria.

#### Hypothesis 1

We expect that the mental health-related quality of life of young informal workers would be relatively low. In the absence of any previous studies evaluating the mental health of young informal construction workers in Nigeria, we wanted to examine the extent to which young informal construction would assess their mental health.

#### Aim 2

To investigate the extent to which and how reciprocity of social support occurs among young informal workers and their social networks despite their socioeconomic conditions.

#### Hypothesis 2

Following previous studies [36, 47, 64] that have found that young informal workers rely heavily on their social networks for health-related needs, we expect that the level of reciprocity among young informal construction workers in Nigeria would be high.

#### Aim 3

 To investigate the relationship between reciprocity and the mental HRQoL of young informal construction workers in Nigeria.

#### Hypothesis 3

Following the outcomes of previous studies on reciprocity and mental health among young people, we expect that young informal construction workers with higher reciprocity scores will report better mental health than those in less reciprocal relationships.

## Methods

### Participants and data collection

This study used a cross-sectional mixed-methods design, including quantitative and qualitative methods. The target population in this study are young informal construction workers (also known as young artisans/artisans in this study) in Nigeria. Given the geographical region under consideration, this study followed the African definition of young people, thereby defining young people, as people between the ages of 18 to 35 years [1]. The present study employed a multistage sampling technique to identify construction sites and select participants, thereby mitigating potential selection bias that could have influenced the research outcomes. The inclusion criteria for the participants were: 1) between 18 to 35 years old; 2) worked as a construction worker for at least 12 months. The questionnaires were self-completed (or completed with the help of a research assistant in case of reading or writing difficulties). A total of 686 (consisting of 633 males and 53 females) and 32 (consisting of 17 males and 15 females) young informal construction workers participated in the quantitative and qualitative study, respectively.

A cross-sectional survey method was employed to collect the quantitative data between September 2021 and January 2022. While in the qualitative study we employed an in-depth interview method to elicit detailed responses that could not be explained by the quantitative survey. The purpose of the qualitative dimension of this study is to understand how reciprocity relates with the health-related quality of life of young construction artisans in Nigeria. The participants of the qualitative interview were selected from the participants of the quantitative survey using a systematic sampling approach, whereby women were prioritised given their small representation in the construction industry (refer to the descriptive statistics in Table 1 to see the gender distribution in the study). Pseudonyms were assigned to the participants for both analytical purposes and ethical considerations.

**Table 1:** Socio-demographic characteristics of study participants

Variables	n	Percentage (%)	Mean	Std. Deviation	Minimum	Maximum
**Age**			26.3	(4.9)		
**Gender**						
Male	633	(92.3)				
Female	53	(7.7)				
**Marital Status**						
Single	456	(66.5)				
Married	230	(32.7)				
**Education**						
None	35	(5.1)				
Primary	73	(10.6)				
Secondary	476	(69.4)				
Tertiary	102	(14.9)				
**Working Hours**		**-**	8.9	1.3	5	18
**Income**						
15,000 and below	312	(45.5)				
15,100-N30,000	248	(36.2)				
30,100-N45,000	62	(9.0)				
45,100 and above	64	(9.3)				
**Religious Affiliation**						
Christianity	339	(49.4)				
Islam	347	(50.4)				
Others	1	(0.1)				
**Workplace safety**						
Feeling Safe	555	(81.0)				
Feeling Unsafe	131	(19.0)				
**NHIS**						
Yes	62	(9.0)				
No	624	(91.0)				

*N* = 686; is the official currency of Nigeria – Naira. 1 = 0.0024 USD/0.019 HKD; *NHIS *National health insurance scheme

### Measures

With the realisation that biomedical measures are insufficient for accurately measuring people’s overall health status, health-related quality of life has gained widespread acceptance as a legitimate health outcomes indicator [15]. Mental health in this study was represented by mental health-related quality of life, and the latest version twelve-item short-form survey commonly known as the SF-12 version 2 (SF-12v2) was used to measure the health-related quality of life of young construction artisans in Nigeria. The permission for this instrument was obtained from Quality Metric Incorporated, LLC (https://www.qualitymetric.com/health-surveys-old/the-sf-12v2-health-survey/), with license number QM056422. The SF-12v2 has been shown to predict at least 90% of the variance in the physical and mental health summary scales derived from the SF-36 [63]. It is therefore an appropriate measure to capture the health status of people when the constraints on questionnaire length is factored [63]. However, the focus of this study is on the mental components summary (MCS) of the SF-12v2 scale.

The MCS scores range from 0 to 100, with higher scores indicating better mental HRQoL. A score of 50 and below on the MCS has been recommended as a cut-off to determine a poor mental condition, moreover, a score of 42 and below may be indicative of clinical depression [63]. The SF-12v2 has been utilized extensively across different populations, but this may likely be the first study to utilize the scale among young informal construction workers in Nigeria. The MCS score was computed using the QualityMetric Patient Reported Outcomes (PRO) scoring software version 2.0 (QualityMetric Incorporated, USA).

The reciprocity scale used to assess the exchange of social support between participants and members of their social networks was adapted from [40]. It consists of 10 items, which are divided into two sets of 5 items each. To ensure its suitability for the study’s context, the scale was modified after a pilot study was conducted to test its validity and reliability. The original questions, such as “How likely would you be there for one or more members of your community/group…?” and “How likely would one or more members of your community group be there for you…?”, were changed to “In the past 12 months, how likely do you support one or more members of your networks…?” and “…how likely do one or more members of your networks support you…?”.

All items were evaluated using a 5-item Likert-type response scale, with 1 indicating “very unlikely” and 5 indicating “very likely.” The mean scores of the responses for the 10 items were calculated to obtain a score for each participant. Higher scores (4 and above) indicated higher levels of reciprocity between the respondent and the members of their social network, while lower scores (2 and below) indicated lower levels of reciprocity. The reciprocity scale displayed optimal structural validity, as evidenced by a Comparative Fit Index (CFI) of 0.89. Additionally, the internal consistency of the reciprocity scale was strong, as indicated by a Cronbach’s alpha coefficient of 0.87, suggesting that the items in the scale effectively measure the intended constructs.

To ensure the accuracy of the relationship between the independent and dependent variable, this study considered various extraneous variables that might distort or bias the observed relationship, potentially leading to inaccurate or misleading conclusions. Based on existing literature and contextual understanding, nine covariates were considered in the regression analyses to control for their potential influence on reciprocity and the mental health-related quality of life of young informal construction workers. The following covariates were considered: age, gender, working hours, marital status, religious affiliation, income, education, national health insurance enrolment, and workplace safety (feeling safe at work). Each covariate was coded in a specific manner to facilitate analysis. Gender was coded as follows: female = 0, male = 1. For education, primary school and below were coded as 0, while secondary school and above were coded as 1. Marital status was coded as single = 0 and married = 1. Participants with religious affiliation were coded as 1, while those without religious affiliation were coded as 0. Income was measured on a weekly basis and categorized into groups ranging from N15,000 and below to N45,000 and above. An income of N15,000 and below was scored as 1, while N45,000 and above was scored as 4, with higher scores indicating better income and lower scores indicating lower income. Workplace safety was assessed using a five-item Likert-type scale, with responses ranging from "feeling very unsafe = 1" to "feeling very safe = 5." Higher scores indicated higher levels of workplace safety, while lower scores indicated lower levels of safety. Working hours were measured based on the average daily working hours. Lastly, NHIS enrolment was coded as "Yes = 1" and "No/don't know = 0". These codes were used in the analyses to reflect either better or poorer conditions for each covariate. By considering and coding these covariates appropriately, the study aimed to account for their potential effects, ensuring that the relationship between the independent and dependent variables could be accurately examined and interpreted.

### Data analysis

The statistical software used for the analysis was IBM SPSS version 26 (IBM® SPSS® Statistics, USA). Descriptive statistics, bivariate correlation and regression analyses were conducted using this software. For the confirmatory factor analysis (CFA), IBM SPSS AMOS version 26 (IBM® SPSS® Statistics, USA) was utilized. The MCS score was computed using the QualityMetric PRO_CoRE solution software (QualityMetric Incorporated, USA).

To address missing data, Little’s test [27] was employed to assess if the missing data were missing completely at random (MCAR). Preliminary data analysis was conducted to identify outliers. Univariate outliers were determined using the Z-score criterion, while bivariate outliers were identified using the Mahalanobis distance criterion [48]. The participants’ socio-demographic characteristics, as well as MCS and reciprocity scores, were analysed using descriptive statistics. Mean and standard deviation were calculated for continuous variables, while frequency and percentage were used for categorical variables. Skewness and kurtosis univariate indices were examined to evaluate the normality of the data.

To assess the association between young informal construction workers' reciprocity and their mental health-related quality of life (HRQoL), Pearson's product-moment correlation coefficient was calculated. Correlational analysis was also conducted to identify variables correlated with HRQOL, which could potentially be included as covariates in the analysis. Finally, a hierarchical regression analysis was performed to examine the effect of reciprocity on the mental health of young informal construction workers in Nigeria.

In the qualitative study, the interviews were audio recorded and subsequently transcribed for analysis purposes. To gain familiarity with the data and enhance understanding of the interview content, both authors engaged in multiple readings of the transcribed data. Additionally, the first author supplemented the transcribed interview data by reviewing the field notes. The analysis of the entire dataset was conducted using an inductive approach, commencing with a semantic coding process. This involved a thorough reading of the data, word by word, to identify and highlight sentences and words that were relevant to the topic under investigation. These identified segments were then coded according to the principles described by Braun & Clarke [9].

## Results

### Socio-demographic characteristics of the study participants

The age of the participants ranged from 18 to 35 years, with a mean age of 26.3 years (standard deviation = 4.9). Among the 686 participants in the study, the majority were males, accounting for 92.3% (633 males), while only 7.7% (53 females) were females. Regarding their marital status, 66.5% (456 participants) were single, and 33.5% (230 participants) were married. Regarding the highest level of education attained by the participants, the majority, comprising 69.4%, had completed secondary education. On the other hand, 5.1% of the participants (35 individuals) did not possess any educational qualifications. The average daily working hours for all artisans were 8.97 h, with a standard deviation of 1.31 h. Income was categorized into four groups as outlined in Table 1. The lowest income bracket was N15,000 (US$32.59) and below per week, while the highest income bracket was N45,100 (US$97.98) and above per week. Among the participants, 45.5% (312 individuals) earned N15,000 (US$32.59) and below weekly, while only 9.3% (64 individuals) earned N45,100 (US$97.98) and above. For a detailed overview of the socio-demographic characteristics of the study participants, please refer to Table 1 below.

### Social support reciprocity and mental health-related quality of life scores

As previously mentioned, the assessment of mental health in this study utilized the SF-12v2 scale, which measures mental health-related quality of life. This measure consists of four dimensions: vitality (VT), social functioning (SF), role emotional (RE), and mental health (MH). The mean mental component summary (MCS) score was calculated to be 51.71, with a standard deviation of 8.48. Analysing the scores based on the 25th percentile, it was found that the artisans represented in the study scored below 44.89 on the MCS. Additionally, 50% of the participants scored below 54.02, while the 75th percentile of the population scored above 58.25 (Table 2). The range of MCS scores varied from a minimum of 20.13 to a maximum of 65.65 (Table 2).

**Table 2:** SF-12v2 mental health summary score

	**VT**	**SF**	**RE**	**MH**	**MCS**
Mean	61.15	47.44	49.47	51.10	51.71
25th Percentile	58.90	39.11	45.89	41.26	44.89
50th Percentile (median)	58.90	48.01	56.28	52.74	54.02
75th Percentile	68.74	56.90	56.28	58.47	58.25
Standard Deviation	8.65	9.89	9.78	9.61	8.48
Min	29.39	21.32	14.70	18.32	20.13
Max	68.74	56.90	56.28	64.21	65.65
N	686	686	686	686	686

Table 3 below shows the descriptive statistics of the reciprocity and mental health-related quality of life scores of the study sample. As mentioned in previous sections, reciprocity represent the independent variable for this study. While the mental health-related characteristics represent the dependent variables. The reciprocity scores were relatively high, with a mean score of 3.9 from a maximum 5, and a standard deviation of 0.65. The summary score of the mental health-related quality of life was slightly above the cutoff score for poor good health, the mean score was 51.5. However, 25% of the participants scored 44.89, indicating poor mental health scores for 1 in every 4 young construction artisans (Table 3 below).

**Table 3:** Reciprocity and mental health scores of young construction Artisans

Variables	Mean	Std. Deviation	Minimum	Maximum
Reciprocity	3.9	.65	1.0	5.0
Mental Health	51.7	8.4	20.13	65.7

Going by the quotes from the qualitative study we uncovered two forms of reciprocity that exist among young informal construction workers, direct and indirect reciprocity. The direct reciprocity is one which happens from person to person (i.e., from person A to person B and vice versa). While indirect reciprocity refers to a type of reciprocity that is based on an individual’s relationship with a third-party. In other words, instead of providing direct assistance to the person in need, a third-party provides assistance on their behalf.

### Direct reciprocity

According to our findings from the qualitative study, most young informal construction workers often provided and received social support including, emotional, financial, and professional support from one another (i.e., from person A to person B, and vice versa). Regarding emotional support, all the participants reported that they usually offered and received support from each other in times of need.

A 19-year-old male carpenter reported:

“*Whenever I feel unhappy, worried or depressed about anything I speak to him (referring to his 18years old male colleague). We are like brothers here, if something is wrong with me, he is the first person I speak to and if something is wrong with him, he comes to me, that is how we help each other…*” (Sam, 19years old, male Carpenter).

Similarly, a 34-year-old female labourer reported:

“*On this site, she (referring to her co-worker) is all I have. If anything is wrong with me, maybe somebody annoys me and I am not happy she is the first person I will share with, because she knows how to listen and encourage me... If anything is wrong with her, she will come to me, and I will do the same for her…*” (Ada, 34 years old, female site labourer)

Likewise, regarding professional support on the job (e.g., helping with tasks on the job, standing in for colleagues when ill), most young artisans reported providing and receiving such support from their colleagues. Below is a quote from a young female artisan:

*“This my friend here now (referring to her co-worker), if I come and there is no work for me, but she has work, we can share her work and at the end of the day we will share the money that comes from it. Even though the money is not so much, it doesn’t leave one person leave one person without work for the day.”* (Grace, 34 years old, female Site Labourer/ Cleaner)

A 23-year-old male tiler also shared how they teach and learn from each other on the job:

*“When it comes to work there is no need to be stingy with information. When my colleague asks for my help, maybe to cut tile or show him how to arrange the tiles, I will show him. Afterall, I don’t know everything, if I also need help, I will ask him, that is the only way we can grow.”* (James, 23years old male Tiler)

Several young artisans also reported engaging in direct exchanges of financial resources to support one another during times of need. For instance, an 18-year-old Carpenter shared how these workers assist each other with money to purchase food or other necessary items. This direct exchange of financial support among the workers highlights their solidarity and willingness to assist one another in meeting basic needs. Below is a quote from one of the participants:

*“If I don’t have money today for transport or food, I can borrow from my friend and he can also borrow from me, whenever we have, we will pay each other. Those type of money is not a problem, it is not more than N500 (US$0.65) or N1000 (US$1.29), we can easily payback.” (Joseph, 18 years old male Carpenter).*

However, regarding the financial support, the artisans believed that even if direct reciprocity takes place, it is not sufficient to meet more advanced needs due to the economic constraints experienced by many of them.

*“We help each other in every way, but the problem is that we are in the same condition, if I have serious accident here (i.e., on the construction site) his money will not be enough to take me to the hospital, that is the problem. For things like buying medicine, yes, he can help me, but if maybe I fall from up, his money cannot cover my hospital bill…”* (Daniel, 25 years old male POP Designer)

Such limitations from direct reciprocity due to the hazardous condition experienced by young informal construction workers caused informal to explore other avenues for giving and receiving support.

### Indirect reciprocity

Due to the insufficiency of direct exchanges between young informal construction workers and their social networks, these informal workers often rely on indirect exchanges, especially for health-related needs. Such indirect exchanges often involve mutual aid (such as borrowing/loaning money) within trade unions/groups, religious associations, and other social groups, to access healthcare, provide childcare, or transportation. These indirect exchanges provided young informal workers with the resources they needed to meet their health-related needs. Two major channels of indirect exchanges were identified from this study, trade groups (professional networks) and religious associations. Most (20 artisans) of the respondents reported being a member of either a professional association or religious association (See Table S1). Through these associations the participants reported exchanging support such as financial support and visitation to ill or needy members.

Below is a quote from a 35-year-old Bricklayer (Mason) explaining how artisans pool resources together to provide support to one another through social participation in trade unions:

*“We have a union where we contribute money every month, if any member needs help maybe to go to the hospital or anything else he can get money from the union…If you don’t contribute, they cannot give you any money, so, I usually contribute because anything can happen, and I will need money.”* (Ahmed, 35 years old Bricklayer)

In addition to the financial support exchanged through the groups, the participants (majorly women) reported that they often visited each other when a member of their union is ill or bereaved to provide emotional support.

“*I am a member of the mothers’ union in church, when I am not feeling well women from the church come to visit me. Sometimes, I may be okay, but I have not gone to church in a while, so they will come to check if I am okay. That is how we usually care for each other.”* (Stella, 27years old, female Site Labourer).

Overall, the artisans believed that indirect reciprocity through group participation helped them to overcome the limitations of direct person-to-person interaction. Below is a quote from a 25-year-old labourer that depicts this:

*It is very important to join this group, if I need serious money who will give me? For example, last week, a log of wood cut my hand seriously, and I had to go for stitching. People here contributed money for me because that is our tradition here… If I waited for one person to give me the money I needed, maybe I would not have gone to the hospital by now”* (Jide, 25years old, male Site Labourer)

### Effect of social support reciprocity on the mental health-related quality of life of young informal construction workers in Nigeria

Hierarchical multiple regression analysis was conducted to examine the relationship between reciprocity and the mental health of young construction artisans in Nigeria. Table 3 presents the coefficients obtained from this regression analysis. The purpose of this analysis was to determine whether reciprocity can predict the mental health of young construction artisans, while controlling for various factors such as age, gender, working hours, marital status, religious affiliation, income, educational qualification, NHIS enrolment, and workplace safety.

After controlling for the aforementioned factors, the results revealed that reciprocity significantly predicted the mental health of young informal construction workers, explaining 28% of the variance (R^2^ = 0.280, *p* < 0.001). This indicates that reciprocity plays a substantial role in influencing the mental health outcomes of these individuals. The regression coefficient associated with reciprocity indicates that for every unit increase in reciprocal scores, the mental health scores of the young construction artisans increased by 2.44 points (Table 4). This effect was statistically significant at a 99.9% confidence interval (CI). Consequently, individuals who engage in higher reciprocal relationships may experience mental health that is approximately two times better than those who have lower levels of reciprocity.

**Table 4:** Hierarchical multiple regression analysis of reciprocity and mental health (MCS-12)

**Variables**	**Model 1**		**Model 2**		
	**B**	**R**^**2**^	**B**	**R**^**2**^	**ΔR**^**2**^
	-	.171		.197	.029
Age	-.256***		.250		
Gender	.737		.515		
Marital Status	-.095		-.270		
Educational Qualification	-1.532**		-1.515**		
Income	1.798***		1,864**		
Working hours	-.533^a^		.502*		
Workplace safety	5.328**		5.134*		
Religious Affiliation	1.827***		1.672**		
NHIS	3.545***		3.696**		
Reciprocity	-	-	2.351***		

*B* Unstandardized beta value

*p < 05

**p < .01

***p < .001

### Reciprocity and mental health: evidence from the qualitative study

The qualitative study provided valuable insights into the influence of reciprocity on the mental health of young informal workers, uncovering the significant impact of both direct and indirect reciprocity on their mental health.

### Direct reciprocity and mental health

This study revealed that direct reciprocity played a significant role in the mental health of young informal workers, as the exchange of emotional support among them positively impacted their well-being. According to the responses from the participants, direct reciprocity helped to produce happiness and ignite hope. For instance, a female artisan shared her experience, highlighting the adverse impact of work instability and irregular payments on her emotional well-being. Despite these challenges, she expressed how the support she receives from her colleagues brings her happiness, regardless of the circumstances she faces. According to her this is the same experience among many female informal construction workers.

*“Our job has a way of frustrating you, some days you will come to work and there is no work. Some other days there’s work but no pay or half pay, those kinds of things are very painful. At that moment I feel very unhappy. If my friend has a job, she can invite me for us to do it together, and at the end of the day we can share the money. For us, it’s not about the money, because it cannot do much, it’s just the feeling that somebody cares, it makes me happy, it brings hope that everything will be fine. Because of that if she is in a similar situation, I will support her anyway I can.”*

Furthermore, some of the participants reported how the emotional support exchanged between co-workers helped to improve their feeling, which may have been dampened by regular shouts and insults hauled on them by their supervisors/employers.

*“Sometimes the way my oga (Boss) will shout at me and insult me you will think I stole his life savings. When it is too much, I cry because it is very sad, it’s work I came to and not correction centre. After he insults me like this, I have to take a break for some time because I cannot work feeling that way… Most times I talk to him (referring to a fellow 19-year-old colleague), and sometimes even if I don’t say anything he (referring to a fellow 19-year-old colleague) can tell that I am not happy, he will find a way to talk to me, or do something to make me laugh. He knows how to make me feel better when I am sad. When something wrong with him, he will come to me too, and I will encourage him. That is how we make each other happy, if not somebody will die from overthinking…”* (Joseph, 18 years old male carpenter)

Our observations indicated that while direct reciprocity through the exchange of emotional support positively contributed to the emotional well-being of young informal construction workers, we also recognized its limitations in addressing their comprehensive mental health concerns. We observed that these workers have needs that encompass both emotional and financial support, and the inability to fulfil either of these needs can worsen their mental health condition. Below is a quote from a 28-year-old male labourer, on how the limitations of relying on individual support led to the loss of his baby, and consequently resulted into depression:

*“I lost my baby because of N100,000 (approximately US$129). I needed money to take her to hospital for surgery, I did not have it and I could not get it from the people around me, it is either they don’t have or what they can give is far from what I need. It took me two months to gather that money, but it was already too late… It makes me sad every time I think of it, I become depressed, because it could have been avoided if the people close had the resources to help.”* (Idris, 28years old male site Labourer)

Several other participants reported how giving or receiving financial support was difficult because of their financial situations as artisans. One of the participants, Daniel, a 25 years old POP designer/Bricklayer said*, “…by the time I receive this money, it is finished… if anybody asks me for help, I cannot give because I don’t even have for myself…”* Therefore, a more holistic approach that addresses both emotional and financial aspects was crucial for effectively supporting the mental health of young informal construction workers. Based on this many young informal construction workers relied on indirect reciprocal approach to meet their needs.

### Indirect reciprocity and mental health of young informal construction workers

The study revealed that indirect reciprocity through religious and professional networks helped to mitigate issues of fear and anxiety among young informal workers through the provision of financial support. First, through participation in religious-related groups, the responses from the interview showed that the mutual exchange of social support among informal workers resulted in a feeling of happiness. One of the respondents (Mike, 28 years old, male Electrician) said:

“*We have a fellowship on site, apart from praying for each other, we support each other financially and even emotionally. This is one of the reasons I come regularly, because it makes me happy and less anxious, knowing that I have people who are there for me*.” (Mike, 28years old, male Electrician)

Another artisan shared that their involvement in the trade union facilitated easier reciprocity and played a significant role in alleviating their fear and anxiety related to accidents and injuries on the construction site.

“…*it is easy to give and also receive help through our union, because we have monthly contributions, and because of this contribution I am not too afraid of being injured. I know that the union will cover my bill since I contribute regularly...*” (Paul, 31years old male Bricklayer)

Furthermore, receiving and giving support through religious participation also gave a sense of belonging and joy to the artisans, which made them feel happy. A quote below from one of the participants depicts this:

“*This time last year, the government demolished our house, and we had nowhere to go to or nobody to turn to, only the church... My brother!! (referring to the interviewer) I have never been sadder or depressed like that... The way they (i.e., the church) rallied to support us, I can never forget… the pastor gave us a new place to stay, the women brough clothes, food, shoes, and different things. They wiped tears from our face, they made us happy, they brought us joy, we did not spend anything. That was when I knew that I am serving a living God, I realised that I have people in church who are like a family to me. Because of that I cannot leave that church, and if anybody is doing something or something is wrong with somebody, I must be there to support them.*” (Stella, 27years old, female Site Labourer)

In addition to the happiness that came with receiving and giving support, some other interviewees reported that the reciprocal relationship going on in the industry as a result of their participation in trade their associations is a source of hope and comfort for them.

“*For me, there is that feeling of hope that if anything happens to me on site, I have people that can help me. Our work can be depressing but knowing that I have people who can help me brings hope and it makes me feel a bit comfortable. Even when I support my colleagues, I am happy to do it because it can be anybody’s turn tomorrow, and the same people will come through for you….*” (Daniel, 25years old, male POP Designer)

In summary, the qualitative study revealed that both direct and indirect reciprocity contribute to the mental health of young informal workers. However, it became evident that the influence of direct reciprocity on their mental health is limited due to the socioeconomic constraints faced by many of these workers. Consequently, indirect reciprocity emerged as a valuable approach that helps address the limitations of direct reciprocity in promoting the mental well-being of young informal workers.

## Discussion

This study aimed to examine the mental health-related quality of life among young informal construction workers in Nigeria, with a specific focus on exploring the relationship between reciprocity and their mental well-being. By shedding light on these dynamics, this research contributes to our understanding of how reciprocity influences the mental health-related quality of life of young informal construction workers in a low- and middle-income country context.

Regarding the mental health of young informal construction workers, the study found that the mean mental health score was 51.7, indicating a slightly higher score than the threshold for good mental health. However, upon further analysis, it was revealed that the 25th percentile of artisans represented in the study scored below 44.89 in the mental component summary score. This indicates a significant proportion (25%) of workers experiencing lower levels of mental health within the study population. Some of the mental health conditions reported by the participants in this study include anxiety, depression, and fear. This finding is consistent with other research on the mental health of construction workers. A systematic review of causes, effects, and interventions for poor mental health among manual and trade workers in the construction industry found that depression, anxiety, and suicide, are more prevalent among construction workers than in the general population [12]. Another study found that young construction workers have higher rates of depression and anxiety [13]. Although the construction industry is known to have a stressful work environment that can negatively affect workers’ mental health [8, 32], we conjecture that other factors such as job insecurity associated with informality may contribute to the fear, anxiety and depression affecting young informal construction workers in Nigeria. This is because informal construction workers are at an inherent disadvantage in terms of job security, benefits, and wages compared to their formal counterparts [13, 35, 61]. This insecurity can cause higher stress levels and mental health problems, resulting in reduced productivity, efficiency, and work quality. Therefore, it is crucial to promote measures that can improve the mental well-being of young informal construction workers.

In relation to the second aim of this study, our hypothesis posited that social support reciprocity among young informal construction workers would be high. The findings from the study supported our hypothesis, revealing a mean social support reciprocity score of 3.9 out of a maximum score of 5. This result indicates a relatively high level of reciprocity among young informal workers. While this finding is consistent with previous studies [24, 25, 49, 53], the qualitative study helped to uncover that reciprocity within the informal sector is a two-pronged approach that involves direct and indirect reciprocity. Previous studies [24, 25, 49, 53] have largely focused on direct person-to-person reciprocal relationships among young people, but this is only scratching the surface of the issue. Given the hazardous nature of construction jobs and the precarious conditions experienced by young informal workers, including factors like low and unstable income, job insecurity, and limited access to social services, relying solely on direct person-to-person support is often insufficient to address their needs effectively. For example, due to their limited income, informal workers may not be able to provide adequate financial support to a co-worker who has experienced an accident, hindering the victim’s ability to access necessary healthcare. According to Jou and Fukada [25] the lack of robust reciprocal relationships (i.e., the insufficiency of support given or received) is associated with poor mental health among young people.

Furthermore, the lack of access to social services among these young informal workers poses an additional challenge, as it diminishes the potential effectiveness of the support they receive or provide to their peers in promoting their mental well-being. Many of these workers also face barriers in accessing vital social services, such as the National Health Insurance Scheme (NHIS), which could make mental health services more affordable and accessible. Consequently, even if they receive financial support from friends or family members, it is often insufficient to meet their healthcare needs. The absence of accessible mental health services makes it challenging for them to cope with issues of stress, depression, or anxiety effectively. Therefore, the challenges faced by these young informal workers have highlighted the need for an alternative approach to the exchange of social support that can help address their problems. As a result, they have adopted an alternative reciprocal approach known as indirect reciprocity. This approach allows for support to be provided and received through indirect means, such as community organizations, online platforms, or non-profit initiatives. By utilizing indirect reciprocity, these workers can access support and resources that may not be readily available through direct person-to-person relationships. This form of reciprocity has been found to be particularly beneficial in situations where access to direct forms of assistance is limited [36].

From our qualitative study we uncovered that young artisans engaged in indirect reciprocity by harnessing their social participation in professional or religious associations. Through such associations, these young informal workers could access and exchange social support (i.e., in the form of financial support, emotional support and other forms of support) with their peers, that would otherwise be unavailable to them due to their precarious economic standing. This type of reciprocity is often considered an important part of the informal worker’s social safety net. They can provide access to healthcare, housing, and other goods and services that could be beneficial to their daily lives. They can also foster a sense of community and belonging, playing a key role in young people’s overall wellbeing.

Regarding the relationship between social support reciprocity and mental health, previous studies [25, 51, 53],Von Dem [60] have examined this relationship and found that reciprocity positively impacts the mental health of young people. However, these studies were conducted in either developed Western or Asian countries creating a geographical gap in the literature that required to be filled. Therefore, in this study we analysed the relationship between social support reciprocity and the mental health of young informal construction workers in Nigeria. Using the quantitative method, we found that social support reciprocity among young informal construction workers positively predicted their mental of health. This result supports our third hypothesis, and it is consistent with the findings from previous studies [25, 51, 53],Von Dem [60] in developed Western and Asian contexts. The result indicates that young informal construction workers in Nigeria who engage in high social support are more likely to experience better mental health than their colleagues in less reciprocal relationships. This is likely because the feeling of being supported and connected to others is linked to lower levels of stress and improved overall wellbeing [17, 25, 34, 51, 59]. It is also likely that the presence of strong social support networks helps to buffer the negative effects of poverty and other socio-economic factors on mental health.

While the quantitative study successfully addressed the geographical gap by affirming the positive relationship between social support reciprocity and the mental health of young individuals in the African context, the qualitative study delved deeper into the intricate dynamics through which social support reciprocity impacts the mental health of young informal workers, unravelling captivating insights. The qualitative study revealed that social support reciprocity among this group is multifaceted, encompassing both direct and indirect forms. Our findings indicated that direct reciprocity played a significant role in promoting mental health, primarily through the exchange of intangible support, such as emotional and work-related assistance. However, given the socio-economic constraints faced by many young informal workers, indirect reciprocity emerged as a crucial mechanism for providing financial support to access healthcare and meet other urgent needs. This form of reciprocity was instrumental in mitigating mental health conditions, including fear, anxiety, and depression, which often arise from the socio-economic distress experienced by young informal workers. It can be likened to a chain of individuals passing a bucket of water to extinguish a fire. While each person alone may not have the ability to put out the fire, their collective action as a group enables them to achieve that goal. Similarly, the collective support and action demonstrated through social support reciprocity among young informal workers proved to be a powerful tool in promoting mental health.

Just like the chain of individuals working together to put out the fire, this form of collective action can drive social change. In the context of our study, it is evident that collaborative efforts and support provided by young informal construction workers through social, religious, or professional groups significantly contribute to the mental wellbeing of young informal workers. This highlights the transformative potential of collective action (i.e., community support and solidarity) in addressing mental health challenges and fostering positive change within this population. By recognizing the significance of indirect reciprocal relationships, this research opens avenues for developing targeted interventions and support programs that address the unique challenges faced by these workers.

### Clinical and policy implication and future research directions

Our study suggests that social support reciprocity among young informal construction workers in Nigeria can contribute to improving their mental health. Since young informal construction workers reported levels of reciprocity could influence their mental health, healthcare professionals should incorporate the assessment of social support reciprocity in their consultation and diagnosis of young people. Where social support reciprocity is lacking or limited, healthcare providers should plan the appropriate interventions. Social support reciprocity is an indicator of the quality of relationships individuals have [6, 7], suggesting that relationship between the individual and his/her social networks is poor. Therefore, healthcare professionals can advise on interventions that can strengthen social relationships rather than relying completely on biomedical measures, which also helps to reduce the financial burden on young informal construction workers who are already economically constrained.

On the policy side, the finding that social support reciprocity among young informal construction workers mostly happens indirectly through social participation in work-related groups suggest that governments can develop targeted interventions and programs that foster social support networks within the construction industry, such as trade associations, mentoring programs, peer support groups, or team-building activities to encourage reciprocal social supports among young informal workers. Such interventions can contribute to an improved mental health and well-being of the general public.

Future research should consider assessing the level of reciprocity between young informal workers and different components of their social networks, such as family, friends, co-workers, to identity which social unit has the most impact on their mental health. Secondly, further research may consider expanding beyond young informal workers in the construction industry to evaluating the mental health of young informal workers in other industries.

### Strengths and limitation

To our knowledge, this is the first study to analyse the reciprocal influence of perceived social support on the mental health of young informal workers in developing countries using a mixed-methods approach. Using a mixed-method approach in this study helped to provide vertical evidence about the different dynamics of the reciprocity and mental health relationship from an African perspective. This evidence is steep and deep as it shows that reciprocity is two pronged and that dynamics of how it influences mental health among young people differs in the developed and developing countries. However, when interpreting our results, a few limitations need to be considered. First, considering that perceived social support reciprocity and mental health are strongly correlated and that the relation between them could be recursive, longitudinal studies should be conducted to determine the direction of the relations. Secondly, our quantitative data is unable distinguish between direct and indirect reciprocity. The purpose of the quantitative data in this study was to ascertain the relationship between reciprocity and mental health as previous scholars have modelled. However, we deployed qualitative evidence to further delineate more detailed dynamics of reciprocity and mental health, which is absent in existing literature. Future studies can move a step further to quantitatively differentiate the impact of direct and indirect reciprocity on the mental health of young people. Thirdly, we cannot exclude the possibility that young informal workers influenced each other during the questionnaire completion, leading to similar results, even though separate completion was recommended. Furthermore, our results cannot be generalized to all developing countries and young informal workers since we only focused on construction workers in Nigeria. Future studies may investigate this relationship in other less developed countries.

## Conclusion

Our study suggests that social support reciprocity positively influences young informal workers' mental health. However, in the African context, the indirect reciprocity through social participation, and not direct reciprocity is what promotes young informal workers mental health. Therefore, policy makers and healthcare professionals should consider these findings when planning interventions to support young people's mental health in developing countries. These interventions should focus on creating and supporting opportunities for social participation, as well as encouraging informal workers to give and receive social support. Such interventions could help to reduce the mental health disparities among young people in African countries.

### Supplementary Information

Supplementary Material 1.

## Data Availability

The datasets generated and/or analysed during the current study are not publicly available due to privacy and ethical restrictions but are available from the corresponding author upon reasonable reques.
