# Supplementary material for: A two-pronged approach to understanding reciprocity and mental health relationship in developing countries: evidence from young informal construction workers in Nigeria
Source: BMC Public Health. 2024 Jul 11;24:1851. doi: 10.1186/s12889-024-19315-x (PMC11238371; doi:10.1186/s12889-024-19315-x)
Supplement: Supplementary file 1 — Supplementary Material 1. [file 12889_2024_19315_MOESM1_ESM.docx]

**Supplementary Material**

***S1. Background Information of Qualitative Study Participants.***

| **S/N** | **Participants** | **Age** | **Gender** | **Marital Status** | **Level of Education** | **Religion** | **Occupation** | **Average weekly income** | **Daily Average Working Hours** | **Member of Professional Association** | **Member of Religious Association** |
| --- | --- | --- | --- | --- | --- | --- | --- | --- | --- | --- | --- |
| 1 | Daniel | 25 | Male | Single | Secondary | Christianity | Plaster of Paris (POP) Designer/Bricklayer | N15,100 -N30,000 | 10 Hrs | Yes | Yes |
| 2 | Ada | 34 | Female | Married | None | Christianity | Site Labourer | N15,000 and below | 10 Hrs | No | Yes |
| 3 | Aisha | 23 | Female | Married | Primary | Muslim | Site Labourer | N15,000 and below | 12 Hrs | No | No |
| 4 | Peace | 27 | Female | Married | Primary | Christianity | Site Labourer | N15,000 and below | 13 Hrs | No | No |
| 5 | Grace | 34 | Female | Married | Secondary | Christianity | Site Labourer | N15,000 and below | 9 Hrs | No | Yes |
| 6 | Tolu | 35 | Female | Married | Primary | Christianity | Site Labourer | N15,000 and below | 13 Hrs | No | Yes |
| 7 | Stanley | 29 | Male | Single | Secondary | Christianity | Tiler | N30,100 - N45,000 | 9 Hrs | Yes | No |
| 8 | Mike | 28 | Male | Single | Secondary | Christianity | Electrician | N30,100 - N45,000 | 7 Hrs | Yes | No |
| 9 | Glory | 30 | Female | Married | Tertiary (National College of Education) | Christianity | Site Labourer | N15,000 and below | 11 Hrs | No | Yes |
| 10 | Faith | 19 | Female | Single | Secondary | Christianity | Salesgirl | N15,000 and below | 9 Hrs | No | No |
| 11 | Sam | 19 | Male | Single | Junior Secondary | Christianity | Carpenter | N15,000 and below | 11 Hrs | No | No |
| 12 | Joseph | 18 | Male | Single | Junior Secondary | Christianity | Carpenter | N15,000 and below | 11 Hrs | No | No |
| 13 | Martin | 28 | Male | Single | Secondary | Christianity | Site Labourer | N15,000 and below | 12 Hrs | No | No |
| 14 | Dorcas | 19 | Female | Single | Secondary | Christianity | Salesgirl | N15,000 and below | 11 Hrs | No | No |
| 15 | James | 23 | Male | Single | Junior Secondary | Christianity | Tiler | N15,100 -N30,000 | 10 Hrs | No | No |
| 16 | Christiana | 21 | Female | Single | Secondary | Christianity | Salesgirl | N15,000 and below | 11 Hrs | No | No |
| 17 | Gana | 25 | Male | Single | Secondary | Christianity | Bricklayer | Above N45,000 | 8 Hrs | Yes | No |
| 18 | Paul | 31 | Male | Married | Primary | Christianity | Bricklayer | N15,100 -N30,000 | 10Hrs | Yes | No |
| 19 | Jane | 27 | Female | Single | Tertiary (University) | Christianity | Salesgirl | N15,100 -N30,000 | 10Hrs | No | No |
| 20 | Emeka | 32 | Male | Married | Primary | Christianity | Welder/Aluminium Work | N15,100 -N30,000 | 10Hrs | No | No |
| 21 | KC | 32 | Male | Married | Secondary | Christianity | Carpenter | N15,100 -N30,000 | 9Hrs | Yes | No |
| 22 | Jide | 25 | Male | Single | Secondary | None | Labourer | N15,000 and below | 5 Hrs | No | No |
| 23 | Idris | 28 | Male | Single | Tertiary (Polytechnic) | Christianity | Labourer | N15,000 and below | 9 Hrs | No | No |
| 24 | Stella | 27 | Female | Married | Secondary | Christianity | Labourer | N15,000 and below | 9Hrs | No | Yes |
| 25 | Prince | 30 | Male | Married | Secondary | Christianity | Carpenter | N30,100 - N45,000 | 8Hrs | Yes | Yes |
| 26 | Chris | 30 | Male | Married | Primary | Christianity | Labourer | N15,100 – N30,000 | 10Hrs | No | Yes |
| 27 | Ahmed | 35 | Male | Married | Secondary | Muslim | Bricklayer | N15,100 - N30,000 | 8Hrs | Yes | No |
| 28 | Abdul | 29 | Male | Married | Primary | Muslim | Bricklayer | N15,100 -N30,000 | 10 Hrs | Yes | No |
| 29 | Kemi | 28 | Female | Married | Secondary | Christianity | Labourer | N15,000 and below | 10 Hrs | No | Yes |
| 30 | Abigail | 27 | Female | Married | Primary | Christianity | Labourer | N15,000 and below | 10 Hrs | No | Yes |
| 31 | Amina | 32 | Female | Married | None | Muslim | Labourer | N15,000 and below | 10Hrs | No | No |
| 32 | Hannah | 30 | Female | Married | Primary | Christianity | Labourer | N15,000 and below | 10 Hrs | No | Yes |
